# Glyphosate-based herbicide metabolic profiles in human urine samples through proton nuclear magnetic resonance analysis

**DOI:** 10.5599/admet.2476

**Published:** 2024-12-08

**Authors:** Preechaya Tajai, Giatgong Konguthaithip, Thanyaphisit Chaikhaeng, Churdsak Jaikang

**Affiliations:** 1 Department of Forensic Medicine, Faculty of Medicine, Chiang Mai University, Chiang Mai, 50200, Thailand; 2 Metabolomic Research Group for Forensic Medicine and Toxicology, Department of Forensic Medicine, Faculty of Medicine, Chiang Mai University, Chiang Mai 50200, Thailand; 3 Faculty of Agriculture, Chiang Mai University, Chiang Mai, 50200, Thailand

**Keywords:** Metabolites, pesticides, toxicokinetic, ^1^H-NMR-based metabolomics

## Abstract

**Background and purpose:**

Glyphosate-based herbicides, extensively utilized worldwide, raise concerns regarding potential human risks due to the detection of glyphosate (GLY) in human body fluids. This study aims to address critical knowledge gaps regarding whether GLY undergoes metabolism in humans, particularly considering the limited information available on human metabolism.

**Experimental approach:**

The study investigated GLY and its metabolites in eight amenity horticultural workers using proton nuclear magnetic resonance (^1^H-NMR) data analysis. Multiple spot urine samples were collected before and after herbicide applications.

**Key results:**

Findings reveal the presence of GLY and its metabolites (AMPA, formaldehyde, sarcosine, glyoxylic acid, and methylamine). Results demonstrate a moderate correlation between median GLY concentration and its metabolites within the studied population.

**Conclusion:**

Persuasive evidence suggests the potential metabolism of GLY in humans. ^1^H-NMR data analysis might be a promising technique for determining the metabolism of GLY in humans, offering valuable insights into urinary excretion patterns.

## Introduction

Glyphosate-based herbicides (GBHs) are the most extensively utilized herbicides worldwide, containing glyphosate (GLY), or N-(phosphonomethyl) glycine, as their active component [1,2]. Despite GBHs being used in 140 countries globally and commanding a corresponding global market share of 71.6 % from 1974 to 2014, evidence regarding their kinetics in humans, especially concerning metabolism, is still lacking [1-3]. Moreover, the toxicity of GBHs has been a subject of continuous debate. While the International Agency for Research on Cancer (IARC) under the World Health Organization (WHO) classified GLY as “probably carcinogenic to humans” (Group 2A), the European Food Safety Authority (EFSA) identified no apparent risk to consumers [4,5].

The extensive use of GBHs has led to their widespread presence in the environment [3]. Consequently, exposure to GBHs has emerged as a significant public health concern [6,7]. Furthermore, both GLY and its major metabolite, aminomethylphosphonic acid (AMPA), have been detected in the environment and various human body fluids, such as urine, serum, and breast milk [6-8]. GLY urinary levels are significantly higher (up to 7.6 μg L^-1^) in regions with extensive use, lower (less than 4 μg L^-1^) among the general population, and even higher (ranging from 0.7 to 292 μg L^-1^) in occupationally exposed populations [7,9,10]. Previous studies have reported that the urinary excretion of GLY follows first-order kinetics, exhibiting a two-phase excretion pattern with a rapid phase half-life of six to nine hours and a slower phase half-life of 18 to 33 hours [2,11-13]. According to the EFSA, approximately 20 % of the ingested GLY is rapidly absorbed in mammals, but the absorbed GLY is poorly metabolized and rapidly eliminated through urine excretion [4]. Nonetheless, the unchanged GLY recovered in urine samples ranged from 1 to 6 %, while the metabolite AMPA was detected at only 0.01 to 0.04 % of the total administered GLY dose [2,13].

Currently, limited information is available regarding human metabolism [2,11-14]. Microbial degradation pathways of GLY have been frequently investigated [15-17]. Various organisms can degrade GLY in nature [16-18]. Furthermore, several studies provide convincing evidence of potential health consequences resulting from microbiome alterations associated with GLY [16,17,19-23]. However, evidence is still lacking regarding human metabolism, specifically whether GLY is metabolized by the human metabolism system, human microbiota, or a combination of both. More evidence regarding the metabolism and fate of GLY in humans is required to perform reliable risk assessments. This information could contribute to raising awareness and encouraging people to minimize exposure, thereby mitigating potential health risks [11,24]. Therefore, the question arises as to whether GLY is metabolized by the human metabolism system, human microbiota, or a combination of both.

**Figure 1.**
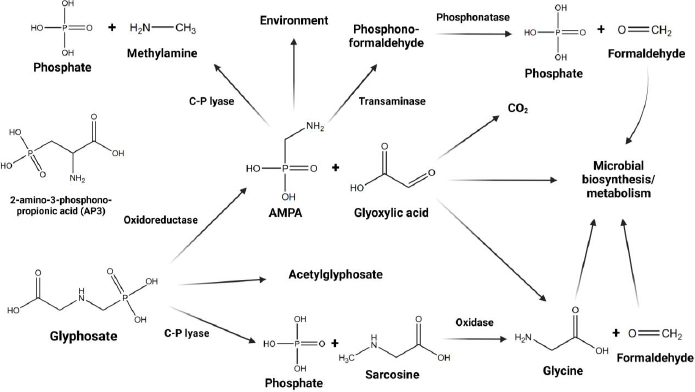
Potential pathways for metabolism and degradation of glyphosate.

GLY degradation follows two primary pathways, as illustrated in Figure 1 [15-17]. In the first pathway, sarcosine accumulates, leading to the subsequent formation of glycine and formaldehyde [15]. The second pathway results in the production of glyoxylic acid and AMPA [15,17], with AMPA further metabolizing to generate methylamine [15]. Consequently, the present study identified not only primary GLY but also various metabolites, including AMPA, formaldehyde, sarcosine, glyoxylic acid, and methylamine, in urine samples. The present study aims to evaluate GLY and its metabolites in amenity horticultural workers through the analysis of proton nuclear magnetic resonance (^1^H-NMR) data from human urine.

The collection and analysis of multiple spot urine samples collected before and after pesticide applications enable the assessment of the appropriateness of the sampling strategy for evaluating exposure to GBHs.

## Experimental

### Reagents

Methanol, chloroform, deuterium oxide (D_2_O), and 3-(trimethylsilyl)-[2, 2, 3, 3-d4]-1-propionate sodium salt (TSP) were purchased from Sigma-Aldrich (St. Louis, MO, USA). All chemicals used were of analytical grade.

### Ethical approval and informed consent

The study was conducted following the Declaration of Helsinki and approved by the Institutional Review Board (Human Ethics Committee), the Faculty of Medicine, Chiang Mai University, Thailand. The ethics approval reference number is No. 180/2022 (Study code: FOR-2564-08598). Eligible participants were those actively engaged as herbicide sprayers, utilizing GBHs during the study period. Written consent was obtained from all participants before their enrollment, and the study results were reported anonymously.

### Study site and study population

This was a prospective study on the kinetics of GLY metabolism conducted in Chiang Mai, Thailand. Healthy volunteers aged between 20 and 60 years were recruited, with a specific focus on herbicide sprayers who applied GBHs to their crops. Additionally, participants refrained from exposure to GBHs, other herbicides, medications, or chemicals for a minimum of 7 days prior to urine collection. All participants meeting these criteria were eligible for inclusion in the study.

Inclusion criteria

1. Herbicide sprayers with documented exposure to GBHs
2. Age between 20 and 60 years
3. Healthy status without underlying diseases

Exclusion criteria

1. Use of herbicide mixtures
2. History of chronic conditions such as diabetes or metabolic disorders
3. Recent (7 days) medical treatments, alcohol consumption, or smoking

### Urine sample collection and preparation

All participants were requested to collect their urinary samples before and up to 72 h after the application of GBHs, using provided plastic bottles. Selection for toxicokinetic analysis was based on participants providing a minimum of four spot urine samples, specifically: (1) urine before the initial spraying task (time 0), (2) urine within 6 h after the first spraying task (time 0 to 6 h), and so forth up to (9) urine between 48 and 54 h after the first spraying task (time 48 to 54 h) or within 48-72 h after the first spraying task (time 48 to 72 h). The concentrations of eliminated GLY and other metabolites in urine samples were plotted every 6 h to demonstrate concentration-time profiles [11,25]. After collecting urine samples, 0.5 mg mL^-1^ of sodium azide (NaN_3_) was added as an anti-microbial agent. The samples were then transferred into 15 mL polypropylene centrifuge tubes and labeled with the subject code, date, and time. These samples were then preserved in an ice box for transportation to the Toxicology Laboratory, Faculty of Medicine, Chiang Mai University, for further analysis. Within 24 h of collection, all samples were stored at -20 °C until laboratory analysis.

The urine samples were suspended with acetonitrile in a 1:1 ratio and mixed for 10 min. Subsequently, the mixture underwent centrifugation at 4000 RPM for 10 min, resulting in the separation of the supernatant, which was then lyophilized. Following this, a solution of 0.6 mL containing 0.1 M TSP in D_2_O was prepared. The levels of metabolites were quantified using NMR at 500 MHz, employing a method to eliminate interference from water resonance.

### Acquisition parameters

The proton NMR spectrum was recorded using a Bruker AVANCE 500 MHz instrument (Bruker, Bremen, Germany) equipped with a Carr–Purcell–Meiboom–Gill (CPMG, RD-90°, (t-180°), n-acquire) pulse sequence for ^1^H-NMR measurements. The spectra were obtained at 27 °C with water suppression pre-saturation. The parameters included 16 scans, a 1 s relaxation decay, a 3.95 s acquisition time, an 8278.146 Hz spectral window, a 0.126 Hz free induction decay (FID) resolution, and a 60.40 μS dwell time. A 90° pulse with 16 signal averages (NSAs) was applied. Baseline and phase correction were conducted using TopSpin 4.0.7 software. Spectra ranging from 0 to 12 ppm, with normalization of data to the total integrated area, were analyzed. Metabolite resonances were identified through human databases [26]. TSP functioned as an internal standard, facilitating the quantification of 24 energy-related metabolites across all samples.

### Internal standard

Trimethylsilyl propanoic acid (TSP) was selected as the internal standard due to its chemical properties. TSP is the chemical of choice because all 14 protons occupy the same chemical environment. This characteristic ensures the signal appears at a specific position at 0 ppm and 500 MHz. Notably, this signal arises from a region with a higher magnetic field intensity compared to other protons. In organic compounds, TSP demonstrates non-reactivity and possesses a low boiling point, making it convenient for extraction from the sample.

### Quality control

Distinct arrangements were established for the quality control samples on the ^1^H-NMR platforms. These preparations were essential to calibrate the system before, during, and after the analysis, ensuring systematic observation and minimizing analytical variations. The quality control samples were prepared by combining and thoroughly mixing an equal amount from each sample. These specimens underwent the same procedural steps as the samples following the previously outlined procedures. The exploration of non-targeted metabolites was conducted using the described methods.

### Peak assignment, chemical identification and proton nuclear magnetic resonance data analysis

Urinary metabolite profile analysis identified the presence of GLY, 2-amino-3-phosphonopropionic acid (AP-3), which shares structural similarities with GLY [27], and metabolites of GLY, including formaldehyde, sarcosine, AMPA, glyoxylic acid, and methylamine, following the potential pathways for the metabolism and degradation of GLY (Figure 1). Each chemical compound was identified through the utilization of the Human Metabolome Database (HMDB) [28] and a previously published paper [29]. The Bruker TopSpin version 4.0.7 software was employed for the analysis of peak acquisition and *J*-coupling values. The interpretation of NMR spectra relied on chemical shift values, which played a crucial role in locating the signal for integration, determining the integrated area beneath the signal, analyzing spin-spin coupling, examining signal patterns, and assessing the coupling constant. It was essential to identify and adjust each peak of every non-targeted metabolite by less than 0.01 in comparison to the HMDB database.

The MestRenova Software [30,31] was employed for data exporting and facilitating spectrum visualization. Median values are utilized for data presentation. Normality was assessed using the Kolmogorov–Smirnov test [31].

### Toxicokinetic analysis

**Figure 2.**
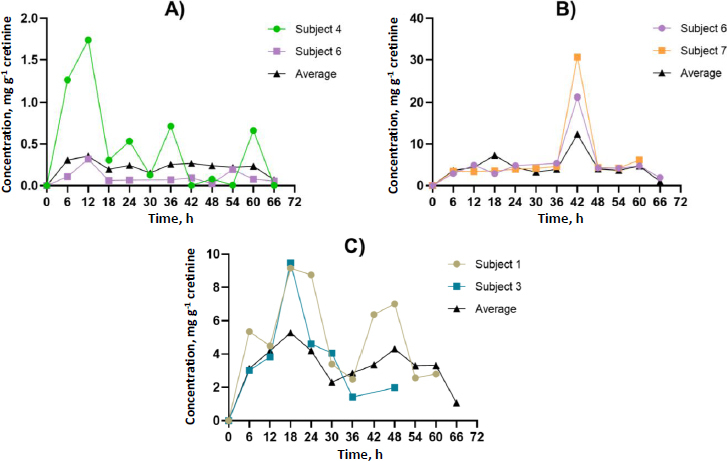
Characteristics of urinary creatinine concentration-time profiles for A - GLY, B - AP-3 and C - the well-known metabolite, AMPA. The concentration-time curves demonstrate the peak time (*T*_max_) in urine, revealing a consistent pattern shared by multiple subjects and aligning with the average concentration in urine.

**Figure 3.**
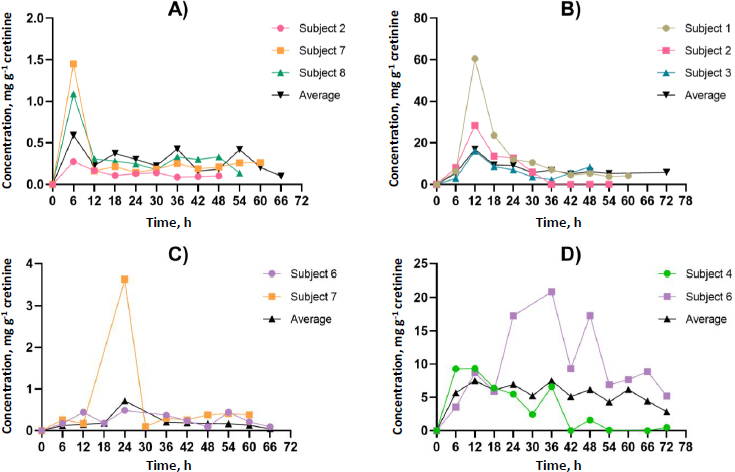
Characteristics of urinary creatinine concentration-time profiles for GLY metabolites, which include A - methylamine, B - formaldehyde, C - glyoxylic acid and D - sarcosine. The concentration-time curves demonstrate the peak time (*T*_max_) in urine, revealing a consistent pattern shared by multiple subjects and aligning with the average concentration in urine. This figure is arranged from the fastest to the slowest peak time.

Participants contributed at least four spot urine samples, ensuring at least two time points occurred after identifying GLY and its metabolites in urine, thereby facilitating toxicokinetic analysis. Figures 2 and 3 illustrate the urinary concentration-time profiles of GLY, AP-3, and its metabolites considered in this study. The PKSolver program [32], employing a non-compartmental model, was utilized to calculate the urinary elimination half-life (*T*_1/2_). The maximum concentration in urine (*C*_max_) represents the highest concentration of GLY and its metabolites excreted over the study duration. The peak time of GLY and its metabolite concentration in urine (*T*_max_) corresponds to the time the body excretes the *C*_max_ (Figures 2 and 3). In brief, the calculation of the area under the curve from 0 to the final time point (*t*) involved the linear trapezoidal method. The area under the curve (AUC) from time 0 to the final time point (*t*) represents the integral of a concentration-time curve between the initial time point 0 and the specified endpoint. In toxicokinetics, the AUC is used to quantify the total exposure of GLY and its metabolites over a specified period. The terminal elimination slope was determined through regression, with emphasis on the largest adjusted *R*^2^, and this regression was based on the last three data points. Urine excretion of GLY and its metabolites was calculated and expressed as concentrations adjusted for creatinine (μg g^-1^ creatinine) over 72 h.

The time point (*t*) is represented in h, and AUC is expressed in μg g^-1^creatinine h^-1^. Creatinine was selected for the normalization of GLY and its metabolites due to its well-established and practical role as a standard normalization factor in urinary metabolomics, particularly in studies involving the measurement of GLY and its metabolites [13,33,34].

### Statistical analysis

Descriptive statistics were used to calculate the demographic characteristics, herbicide application equipment, work practices, and personal protective equipment (PPE) use among the 8 participants. The data were presented as the number of participants and the mean ± standard deviation (SD). Statistical analysis was performed using GraphPad Prism version 8.3.0 for Windows [35,36]. Additionally, a box plot was generated using SRPLOT [37].

## Results

Demographic characteristics, herbicide application equipment, work practices, and the use of personal protective equipment (PPE) among the eight participants are shown in Table 1. All participants were male (100 %), with an average age of 43 ± 15 years, ranging from 24 to 60 years. The equipment used for GBHs application was predominantly the high-pressure lance sprayer backpack (50 %).

**Table 1.** Demographic characteristics, herbicide application equipment, along with work practices and personal protective equipment (PPE) use among the 8 male participants, mean age 43 (SD = 15)

Variable	Number of participants	Share, %
Equipment		
High-pressure lance sprayer, separated	2	25
High-pressure lance sprayer backpack	4	50
Manual lever-operated knapsack sprayer	2	25
Work practices		
Mix and load GBHs	8	100
Handle the equipment	8	100
Clean and collect the equipment	8	100
PPE use		
Use a mask or respirator	0	0
Wear rubber gloves	2	25
Wear rubber boots	6	75

All participants were involved in mixing and loading GBHs, as well as handling, cleaning, and collecting the equipment themselves. Unfortunately, none of the participants used a mask or respirator (0 %), only 25 % wore rubber gloves, and 75 % wore rubber boots as part of their PPE.

### GBHs metabolic profiles

**Figure 4.**
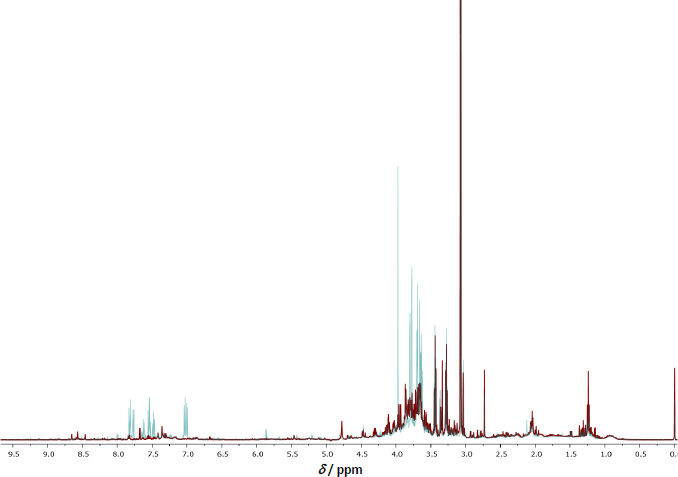
Results of NMR-based metabolomics analysis. The 500 MHz ^1^H-NMR spectra of the urine sample obtained from subject number 1 are presented. The chromatogram overlay highlights distinctions between the samples collected before the application of GBHs, depicted in red, and those obtained 12 h post-application, depicted in blue.

The urinary metabolite profile analysis revealed the presence of GLY, AP-3 (which shares structural similarities with GLY), and metabolites of GLY in samples collected from eight agricultural workers. The sampling occurred at various intervals: initially before the application of GBHs and subsequently at 6-hour intervals, extending up to 72 h post-application. The urinary metabolite composition was elucidated through a 500 MHz ^1^H-NMR chromatogram, as illustrated in Figure 4.

Identification of metabolites was achieved by referencing the Human Metabolome Database (HMDB) [28][ref.]. The chemical compounds detected in the urine samples from the agricultural workers included not only the primary GLY and its structurally similar counterpart AP-3 but also several metabolites such as AMPA, formaldehyde, sarcosine, glyoxylic acid, and methylamine. This comprehensive analysis provides valuable insights into the urinary excretion of GLY and its metabolites following exposure, shedding light on the metabolic pathways and kinetics associated with the application of GBHs in agricultural settings.

### Toxicokinetic profiles

**Figure 5.**
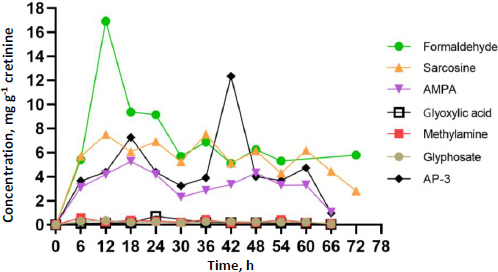
Urinary creatinine concentration-time profiles of the average concentration of GLY, AP-3, and GLY metabolites, including methylamine, formaldehyde, glyoxylic acid, and sarcosine, collected from eight agricultural workers.

The dataset comprised 13 data points for each participant, representing individual spot urine samples collected at different time points over a 72-hour period. Participants contributed a minimum of four spot urine samples, with at least two time points occurring after the detection of GLY and its metabolites in urine, thereby facilitating toxicokinetic analysis. The toxicokinetic evaluation of GLY and its metabolites, including formaldehyde, sarcosine, AMPA, glycoxylic acid, and methylamine, unveiled unique patterns in both time-related and concentration dynamics. Toxicokinetic parameters and urinary concentration-time profiles are presented in Table 2 and Figure 5. The medians of elimination half-life (*T*_1/2_) are arranged in ascending order from the most rapid to the most prolonged: methylamine (5.8 h), AP-3 (8.5 h), sarcosine (10.6 h), AMPA (11.9 h), glyoxylic acid (13.4 h), GLY (15.5 h), and formaldehyde (45.3 h). Methylamine demonstrated the shortest half-life at 5.8 h, underscoring its rapid metabolism. Regarding time to reach maximum concentration (*T*_max_), each compound exhibited unique absorption patterns, underscoring diverse rates of absorption and distribution. The medians of *T*_max_ were shown, ranging from the most rapid to the most prolonged: methylamine (6.0 h), formaldehyde, and GLY (12.0 h), AMPA (18.0 h), glyoxylic acid (24.0 h), sarcosine (36.0 h), and AP-3 (42.0 h). The maximum concentrations (*C*_max_) exhibited significant variation, ranging from 16.9 μg g^-1^ creatinine for formaldehyde to 0.4 μg g^-1^ creatinine for GLY. These disparities emphasize the unique bioavailability of each compound. The medians of *C*_max_ (μg g^-1^ creatinine) were shown, ranging from the highest to lowest: formaldehyde (16.9), AP-3 (12.4), sarcosine (7.5), AMPA (5.3), glyoxylic acid (0.7), methylamine (0.6), and GLY (0.4). Given the unknown exposure doses in occupational settings, which could occur through dermal and inhalation routes, this study employed the PKSolver program to predict the overall exposure doses. Analysis of the area under the curve (AUC 0-t) values provided insights into the comprehensive exposure profiles of each compound over time. Notably, formaldehyde exhibited the highest AUC at 504.5 μg g^-1^ creatinine h^-1^, emphasizing its prolonged presence. In contrast, glycoxylic acid and GLY demonstrated the lowest AUC values of 15.0 μg g^-1^ creatinine h^-1^, indicating faster elimination. The medians of AUC (μg g^-1^ creatinine h^-1^) were shown, ranging from the highest to the lowest: formaldehyde (504.5), sarcosine (399.5), AP-3 (312.4), AMPA (276.5), methylamine (19.0), GLY (15.0), and glyoxylic acid (15.0). This comprehensive analysis of toxicokinetic parameters enhances our understanding of the absorption, distribution, and elimination characteristics of GLY and its metabolites. Additionally, Tables S1 to S8 in the Supplementary material present the toxicokinetic parameters of GLY and its metabolites for individual subjects.

**Table 2.** Toxicokinetic parameters of urinary GLY and its metabolites.

Kinetic parameters	Formaldehyde	Sarcosine	AMPA	Glyoxylic acid	Methylamine	GLY	AP-3
*T*_1/2_ / h	45.3	10.6	11.9	13.4	5.8	15.5	8.5
*T*_max_ / h	12.0	36.0	18.0	24.0	6.0	12.0	42.0
*C*_max_ /μg g^-1^_creatinine_	16.9	7.5	5.3	0.7	0.6	0.4	12.4
AUC_0-t_, μg g^-1^_creatinine_ h^-1^	504.5	399.5	276.5	15.0	19.0	15.0	312.4
*R* ^2^	0.8	1.0	0.9	0.9	1.0	0.8	0.9

AMPA - aminomethylphosphonic acid; AP-3-2-amino-3-phosphonopropionic acid; AUC_0-t_ - area under the curve from 0 to the final time point (t); *C*_max_ - maximum concentration in urine; GLY, glyphosate; *R*^2^ - correlation coefficient; *T*_1/2_ - elimination half-life; *T*_max_ - peak time of GLY and its metabolites concentration in urine

### Association of GBHs exposure with its metabolites in urine

**Figure 6.**
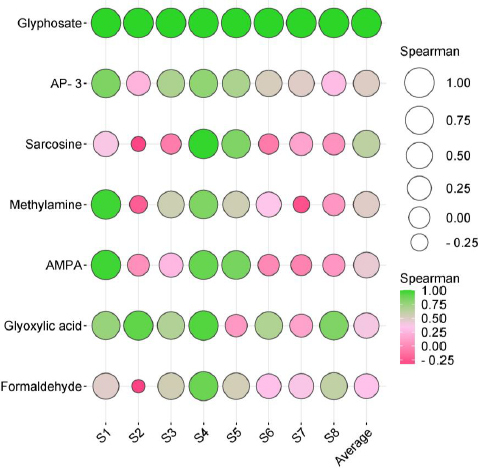
Spearman rank correlation coefficients manifested the subsequent associations between GYL and other metabolites, with GLY as the reference for comparative analysis.

This study evaluated the correlation between GLY and its metabolites using Spearman rank correlation. This statistical method was utilized to investigate whether variations in GLY concentration corresponded to a consistent pattern in the levels of its metabolites. The results exhibited a significant yet moderate correlation between the median GLY concentration and its metabolites within the studied population. The Spearman rank correlation coefficients were as follows: GYL and sarcosine (population correlation coefficient (*ρ*) = 0.62), GLY and AP-3 (*ρ* = 0.50), GLY and methylamine (*ρ* = 0.48), GLY and AMPA (*ρ* = 0.44), GLY and glyoxylic acid (*ρ* = 0.38), GLY and formaldehyde (*ρ* = 0.32), as depicted in Figure 6 and detailed in Table S9.

Notably, the profile of GLY and its metabolites in individual subjects (S1-S8) revealed discernible inter-individual variations in metabolism and/or elimination processes. These observations provide insight into potential associations between individual disparities in metabolism or elimination and the diverse concentrations of GLY and its metabolites.

## Discussion

For decades, several international agencies have debated the health impact of GBHs, particularly on the endocrine and reproductive systems [17,38]. The widespread use of GBHs has resulted in their widespread occurrence in the environment [3]. This has raised public health concerns, as both GLY and its metabolite, AMPA, have been detected in various human body fluids, confirming human exposure [6-8]. In occupationally exposed populations, GLY urinary levels are significantly higher compared to other exposed populations [7,10,25]. Currently, limited information is available regarding the metabolism in humans [2,11-14]. Microbial degradation pathways of GLY have been frequently investigated [15-17]. Furthermore, there is considerable evidence regarding the toxicity of GLY on the human microbiome [16,17,19,20]. However, there still lacks evidence regarding human metabolism, specifically whether GLY is metabolized by the human metabolism system, human microbiota, or a combination of both. The EFSA summarized that in mammalians the absorbed GLY is poorly metabolized and rapidly excreted via the urine, showing no potential for bioaccumulation [4]. Many studies have shown that the major metabolite of GLY is only AMPA, and limited evidence has been found to measure other metabolites [2,10,11,24,39]. GLY degradation follows two primary pathways, as illustrated in Figure 1 [15-17]. In the first pathway, sarcosine accumulates, and the GLY C-P bond undergoes dephosphorylation by C-P lyase [15]. Subsequently, sarcosine undergoes metabolism through oxidase, resulting in the formation of glycine and formaldehyde [15]. In the second pathway, the C-N bond is cleaved by the enzyme GLY oxidoreductase, leading to the production of glyoxylate and AMPA [15, 17]. AMPA then undergoes metabolism via C-P lyase, generating methylamine [15]. The present study identified not only the primary GLY and its structurally similar counterpart AP-3 but also several metabolites, including AMPA, formaldehyde, sarcosine, glyoxylic acid, and methylamine, within urine samples collected from agricultural workers. To the best of our knowledge, the discovery of these additional GLY metabolites, which have not been previously explored, adds a compelling dimension to our understanding. Furthermore, this comprehensive analysis is the first study employing ^1^H-NMR data analysis to determine GLY metabolites, offering valuable insights into the urinary excretion patterns of GLY and its metabolites after exposure in agricultural settings. In light of our findings, there is persuasive evidence suggesting the potential metabolism of GLY in humans. Nevertheless, additional investigations are imperative to substantiate the precise pathways involved.

Various organisms, including numerous bacteria such as *Agrobacterium* spp., *Arthrobacter* spp., GLP-1, *Geobacillus caldoxylosilyticus* T20, *Pseudomonas* spp., *Rhizobium* spp., *Bacillus megaterium* 2BLW, and *Alcaligenes* spp., can degrade GLY in nature [16-18]. Several studies provide convincing evidence of potential health consequences resulting from microbiome alterations associated with GLY, as observed in animal studies [17,21-23]. Additionally, substantial evidence exists regarding the toxicity of GLY on the human microbiome, impacting various regions, such as the microbiota-gut-brain axis, as well as other human microbiomes in the skin, airway, and genital tracts [17,19-21]. Our findings suggest that the human microbiome might play a role in GLY metabolism. However, further studies are still required with a larger sample size and conducted in other settings. Our results are not consistent with a study that investigated the metabolism of GLY by the human fecal microbiota *in vitro*. The samples were collected from 15 volunteers and incubated anaerobically with pure GLY. The results showed no evidence of GLY degradation or the formation of AMPA, a known soil microbial metabolite. This suggests that the human gut microbiome is unable to metabolize GLY [8]. This can be attributed to the well-established understanding that substantial inter-individual variations exist in the composition of the human microbiota [8,17,40]. Several life factors contribute to the uniqueness of an individual’s microbiome, including aspects such as age, diet, underlying diseases, and lifestyle choices such as exercise frequency [41-43]. Another factor that may influence outcomes is the route of exposure. In occupational settings, the primary routes of exposure should predominantly include inhalation and dermal contact, while in the general population, exposure is typically through the ingestion of contaminated GLY [2,8,11]. Our findings demonstrated a moderate correlation between median GLY concentration and its metabolites, consistent with previous human experiments [2]. However, individual subject profiles (depicted in Figure 6 and detailed in Table S9) revealed distinct inter-individual variations in metabolism, suggesting that certain individuals may exhibit varying sensitivity to GLY toxicity.

This study revealed that formaldehyde demonstrated the highest AUC at 504.5 μg g^-1^ creatinine h^-1^, underscoring its prolonged presence. Consistent with the medians of *C*_max_, formaldehyde exhibited the highest value at 16.9 μg g^-1^ creatinine. Formaldehyde is commonly found in GBHs formulations as an impurity, with a maximum amount of 1.3 g kg^-1^ [44-46]. Furthermore, formaldehyde is a metabolite produced through the microbial metabolism pathway of GLY [15,16]. Considering that the highest predicted exposure concentration is associated with formaldehyde, it might be useful as a promising biomarker for measuring exposure to GBHs. This potential is particularly relevant when considered alongside the observed low levels of GLY and AMPA in human biofluids [13]. Given these low levels in human biofluids, the application of highly sensitive techniques becomes crucial for the precise determination of GLY metabolites. Notably, our study indicates that ^1^H-NMR data analysis could be a promising technique, referring to the success of prior investigations [31,47]. In light of our findings, compelling evidence points toward the potential metabolism of GLY in humans. However, further investigations are imperative to substantiate the precise pathways involved. Future studies should involve the collection of human fecal samples from a larger population, followed by the incubation of human fecal suspension samples with GLY under strictly anaerobic conditions. The subsequent measurement of GLY metabolites using the ^1^H-NMR technique will provide valuable insights for a more comprehensive understanding.

## Conclusions

In conclusion, this study represents the first investigation into GLY metabolism in humans. Analyzing eight amenity horticultural workers using ^1^H-NMR data analysis, we identified the presence of GLY and its metabolites (AMPA, formaldehyde, sarcosine, glyoxylic acid, and methylamine). Spearman rank correlation revealed a moderate correlation between median GLY concentration and its metabolites in the studied population. The discovery of previously unexplored GLY metabolites enhances our understanding. This comprehensive analysis, utilizing ^1^H-NMR data, provides valuable insights into urinary excretion patterns post-agricultural exposure. Persuasive evidence suggests potential GLY metabolism in humans, emphasizing the need for additional investigations to substantiate precise pathways. The application of ^1^H-NMR for GLY metabolite measurement holds promise for a more comprehensive understanding.

## Supplementary material

Additional data are available at https://pub.iapchem.org/ojs/index.php/admet/article/view/2476, or from the corresponding author on request.


