# Supplementary material for: Glyphosate-based herbicide metabolic profiles in human urine samples through proton nuclear magnetic resonance analysis
Source: ADMET DMPK. 2024 Dec 8;12(6):957–70. doi: 10.5599/admet.2476 (PMC11661807; doi:10.5599/admet.2476)
Supplement: Supplementary file 2 [file ADMET-12-2476-S1.docx]

*ADMET & DMPK* *12(6) (2024) S19-S24*

*
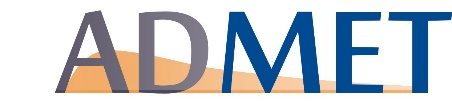
***Open Access : ISSN : 1848-7718**[***http://www.pub.iapchem.org/ojs/index.php/admet/index***](http://www.pub.iapchem.org/ojs/index.php/admet/index)

Supplementary material to

**Glyphosate-based herbicide metabolic profiles in human urine samples through proton nuclear magnetic resonance analysis**

Preechaya Tajai^1,2^, Giatgong Konguthaithip^1,2^, Thanyaphisit Chaikhaeng^3^ and Churdsak Jaikang^1,2^

*^1^Department of Forensic Medicine, Faculty of Medicine, Chiang Mai University, Chiang Mai, 50200, Thailand*

*^2^Metabolomic Research Group for Forensic Medicine and Toxicology, Department of Forensic Medicine, Faculty of Medicine, Chiang Mai University, Chiang Mai 50200, Thailand
^3^Faculty of Agriculture, Chiang Mai University, Chiang Mai, 50200, Thailand*

ADMET & DMPK **12(6)** (2024) 957-970; <https://doi.org/10.5599/admet.2476>

Abbrevations used in Tables S1 to S9:
AMPA - aminomethylphosphonic acid

AP-3 - - 2-amino-3-phosphonopropionic acid

GLY - glyphosate

*T*_1/2_ - elimination half-life

*T*_max_ - peak time of GLY and its metabolites concentration in urine

*C*_max_ - maximum concentration in urine

AUC_0-_*_t_* - area under the curve from 0 to the final time point *t*

*R*^2^ - correlation coefficient

**Table S1.** Toxicokinetic parameters of urinary GLY and its metabolites in Subject 1.

| Kinetic parameters | Formal-dehyde | Sarcosine | AMPA | Glyoxylic acid | Methyl-amine | GLY | AP-3 |
| --- | --- | --- | --- | --- | --- | --- | --- |
| *T*_1/2_ / h | 17.0 | 35.8 | 12.0 | 5.2 | 20.2 | 26.8 | 29.2 |
| *T*_max_ / h | 12.0 | 12.0 | 18.0 | 18.0 | 18.0 | 18.0 | 18.0 |
| *C*_max_ / µg g^-1^ creatinine | 60.5 | 10.4 | 9.2 | 0.2 | 0.4 | 0.2 | 26.4 |
| AUC_0-t_, µg g^-1^ creatinine h^-1^ | 815.3 | 364.6 | 305.6 | 1.8 | 12.2 | 2.7 | 410.5 |
| *R*^2^ | 1.0 | 0.8 | 0.9 | 1.0 | 0.9 | 0.5 | 0.9 |

**Table S2.** Toxicokinetic parameters of urinary GLY and its metabolites in Subject 2.

| Kinetic parameters | Formal-dehyde | Sarcosine | AMPA | Glyoxylic acid | Methyl-amine | GLY | AP-3 |
| --- | --- | --- | --- | --- | --- | --- | --- |
| *T*_1/2_ / h | 4.2 | 14.7 | 19.8 | 7.7 | 34.0 | 8.4 | 501.6 |
| *T*_max_ / h | 12.0 | 6.0 | 24.0 | 42.0 | 6.0 | 42.0 | 30.0 |
| *C*_max_ / µg g^-1^ creatinine | 28.4 | 4.3 | 2.6 | 0.4 | 0.3 | 1.1 | 2.3 |
| AUC_0-t_, µg g^-1^ creatinine h^-1^ | 414.5 | 122.5 | 85.7 | 7.7 | 6.3 | 19.1 | 94.7 |
| *R*^2^ | 0.9 | 0.9 | 0.9 | 1.0 | 0.8 | 0.6 | 0.6 |

**Table S3.** Toxicokinetic parameters of urinary GLY and its metabolites in Subject 3.

| Kinetic parameters | Formal-dehyde | Sarcosine | AMPA | Glyoxylic acid | Methyl-amine | GLY | AP-3 |
| --- | --- | --- | --- | --- | --- | --- | --- |
| *T*_1/2_ / h | 29.5 | 53.6 | 12.6 | 13.7 | 27.2 | 7.1 | 35.3 |
| *T*_max_ / h | 12.0 | 24.0 | 18.0 | 48.0 | 6.0 | 6.0 | 12.0 |
| *C*_max_ / µg g^-1^ creatinine | 16.1 | 9.1 | 9.5 | 0.2 | 1.3 | 0.7 | 9.5 |
| AUC_0-t_, µg g^-1^ creatinine h^-1^ | 302.3 | 282.9 | 174.3 | 5.8 | 22.6 | 21.4 | 234.3 |
| *R*^2^ | 0.5 | 0.4 | 0.9 | 0.6 | 0.6 | 0.8 | 0.6 |

**Table S4.** Toxicokinetic parameters of urinary GLY and its metabolites in Subject 4.

| Kinetic parameters | Formal-dehyde | Sarcosine | AMPA | Glyoxylic acid | Methyl-amine | GLY | AP-3 |
| --- | --- | --- | --- | --- | --- | --- | --- |
| *T*_1/2_ / h | 5.5 | 2.4 | 6.8 | 9.3 | 4.4 | 8.2 | 5.6 |
| *T*_max_ / h | 24.0 | 12.0 | 18.0 | 6.0 | 24.0 | 12.0 | 24.0 |
| *C*_max_ / µg g^-1^ creatinine | 11.3 | 9.3 | 5.4 | 0.2 | 0.3 | 1.7 | 8.5 |
| AUC_0-t_, µg g^-1^ creatinine h^-1^ | 181.6 | 247.8 | 126.0 | 3.9 | 9.1 | 32.6 | 190.3 |
| *R*^2^ | 0.9 | 1.0 | 0.8 | 0.8 | 1.0 | 0.6 | 1.0 |

**Table S5.** Toxicokinetic parameters of urinary GLY and its metabolites in Subject 5.

| Kinetic parameters | Formal-dehyde | Sarcosine | AMPA | Glyoxylic acid | Methyl-amine | GLY | AP-3 |
| --- | --- | --- | --- | --- | --- | --- | --- |
| *T*_1/2_ / h | 51.8 | 53.3 | 24.2 | 26.8 | 8.8 | 12.5 | 14.3 |
| *T*_max_ / h | 48.0 | 30.0 | 24.0 | 18.0 | 36.0 | 48.0 | 48.0 |
| *C*_max_ / µg g^-1^ creatinine | 15.5 | 4.5 | 2.8 | 0.1 | 1.9 | 0.6 | 5.8 |
| AUC_0-t_, µg g^-1^ creatinine h^-1^ | 478.4 | 146.1 | 72.8 | 1.4 | 20.0 | 11.7 | 159.3 |
| *R*^2^ | 1.0 | 0.7 | 0.8 | 0.9 | 0.7 | 0.8 | 1.0 |

**Table S6.** Toxicokinetic parameters of urinary GLY and its metabolites in Subject 6.

| Kinetic parameters | Formal-dehyde | Sarcosine | AMPA | Glyoxylic acid | Methyl-amine | GLY | AP-3 |
| --- | --- | --- | --- | --- | --- | --- | --- |
| *T*_1/2_ / h | 111.1 | 22.9 | 14.9 | 5.2 | 3.1 | 6.6 | 8.8 |
| *T*_max_ / h | 36.0 | 36.0 | 48.0 | 24.0 | 54.0 | 12.0 | 42.0 |
| *C*_max_ / µg g^-1^ creatinine | 15.4 | 20.9 | 9.0 | 0.5 | 2.0 | 0.3 | 21.2 |
| AUC_0-t_, µg g^-1^ creatinine h^-1^ | 474.2 | 767.6 | 283.8 | 18.6 | 33.5 | 6.3 | 368.1 |
| *R*^2^ | 0.5 | 0.8 | 1.0 | 1.0 | 1.0 | 1.0 | 0.9 |

**Table S7.** Toxicokinetic parameters of urinary GLY and its metabolites in Subject 7.

| Kinetic parameters | Formal-dehyde | Sarcosine | AMPA | Glyoxylic acid | Methyl-amine | GLY | AP-3 |
| --- | --- | --- | --- | --- | --- | --- | --- |
| *T*_1/2_ / h | 31.5 | 24.8 | 14.9 | 31.8 | 62.3 | 10.7 | 8.6 |
| *T*_max_ / h | 18.0 | 48.0 | 48.0 | 24.0 | 6.0 | 30.0 | 42.0 |
| *C*_max_ / µg g^-1^ creatinine | 12.0 | 9.5 | 9.0 | 3.6 | 1.5 | 0.2 | 30.7 |
| AUC_0-t_, µg g^-1^ creatinine h^-1^ | 462.6 | 438.4 | 283.8 | 45.6 | 19.2 | 3.4 | 392.8 |
| *R*^2^ | 0.9 | 0.9 | 1.0 | 0.3 | 0.3 | 0.6 | 0.7 |

**Table S8.** Toxicokinetic parameters of urinary GLY and its metabolites in Subject 8.

| Kinetic parameters | Formal-dehyde | Sarcosine | AMPA | Glyoxylic acid | Methyl-amine | GLY | AP-3 |
| --- | --- | --- | --- | --- | --- | --- | --- |
| *T*_1/2_ / h | 26.9 | 23.2 | 28.0 | 12.2 | 16.3 | 27.0 | 13.3 |
| *T*_max_ / h | 24.0 | 12.0 | 12.0 | 18.0 | 6.0 | 24.0 | 18.0 |
| *C*_max_ / µg g^-1^ creatinine | 11.3 | 11.3 | 9.3 | 0.7 | 1.1 | 0.5 | 10.8 |
| AUC_0-t_, µg g^-1^ creatinine h^-1^ | 299.9 | 313.9 | 226.7 | 18.0 | 18.8 | 10.5 | 261.7 |
| *R*^2^ | 0.6 | 1.0 | 1.0 | 0.9 | 0.8 | 0.4 | 1.0 |

**Table S9.** The Spearman rank correlation coefficients manifested the subsequent associations between GYL and other metabolites, with GLY as the reference for comparative analysis.

|  | Subject number | | | | | | | | |
| --- | --- | --- | --- | --- | --- | --- | --- | --- | --- |
|  | 1 | 2 | 3 | 4 | 5 | 6 | 7 | 8 | Average |
| Glyphosate | 1.00 | 1.00 | 1.00 | 1.00 | 1.00 | 1.00 | 1.00 | 1.00 | 1.00 |
| AP-3 | 0.84 | 0.24 | 0.68 | 0.78 | 0.69 | 0.52 | 0.48 | 0.29 | 0.50 |
| Sarcosine | 0.37 | -0.31 | -0.07 | 0.99 | 0.83 | -0.07 | 0.15 | 0.05 | 0.62 |
| Methylamine | 0.98 | -0.22 | 0.56 | 0.84 | 0.55 | 0.35 | -0.27 | 0.07 | 0.48 |
| AMPA | 0.98 | 0.03 | 0.26 | 0.90 | 0.86 | 0.00 | -0.03 | 0.07 | 0.44 |
| Glyoxylic acid | 0.76 | 0.92 | 0.66 | 0.95 | 0.08 | 0.67 | 0.14 | 0.83 | 0.38 |
| Formaldehyde | 0.48 | -0.32 | 0.55 | 0.89 | 0.54 | 0.31 | 0.37 | 0.61 | 0.32 |


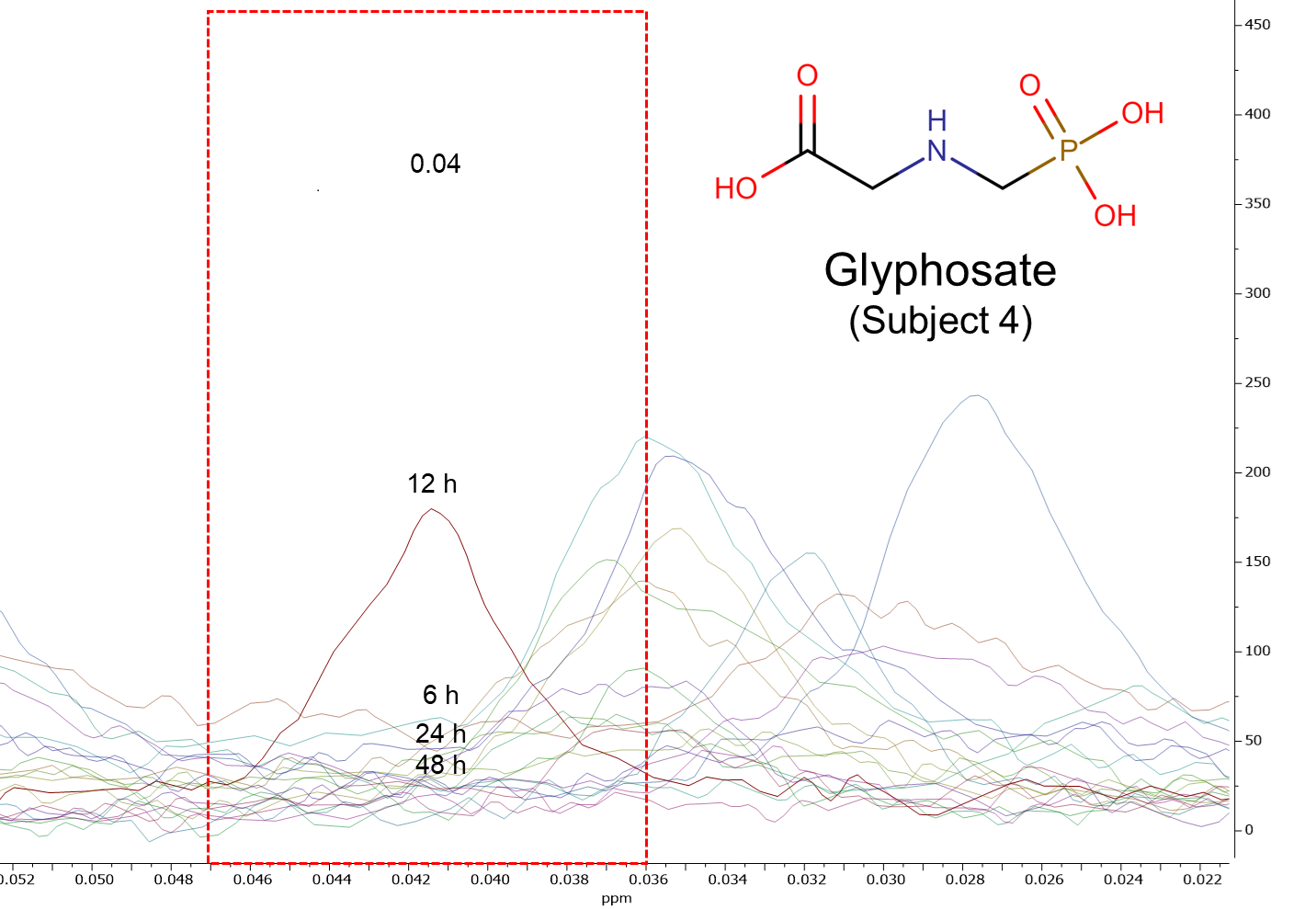


*δ* / ppm

**Figure S1.** Results of NMR-Based Metabolomics Analysis. The 500 MHz ¹H-NMR spectra of glyphosate in urine samples from subject number 4 are presented. The chromatogram overlay highlights distinctions between samples collected at different time points, demonstrating changes in the metabolite profile over time. The structure of glyphosate was obtained from the Human Metabolome Database (<https://hmdb.ca/>).


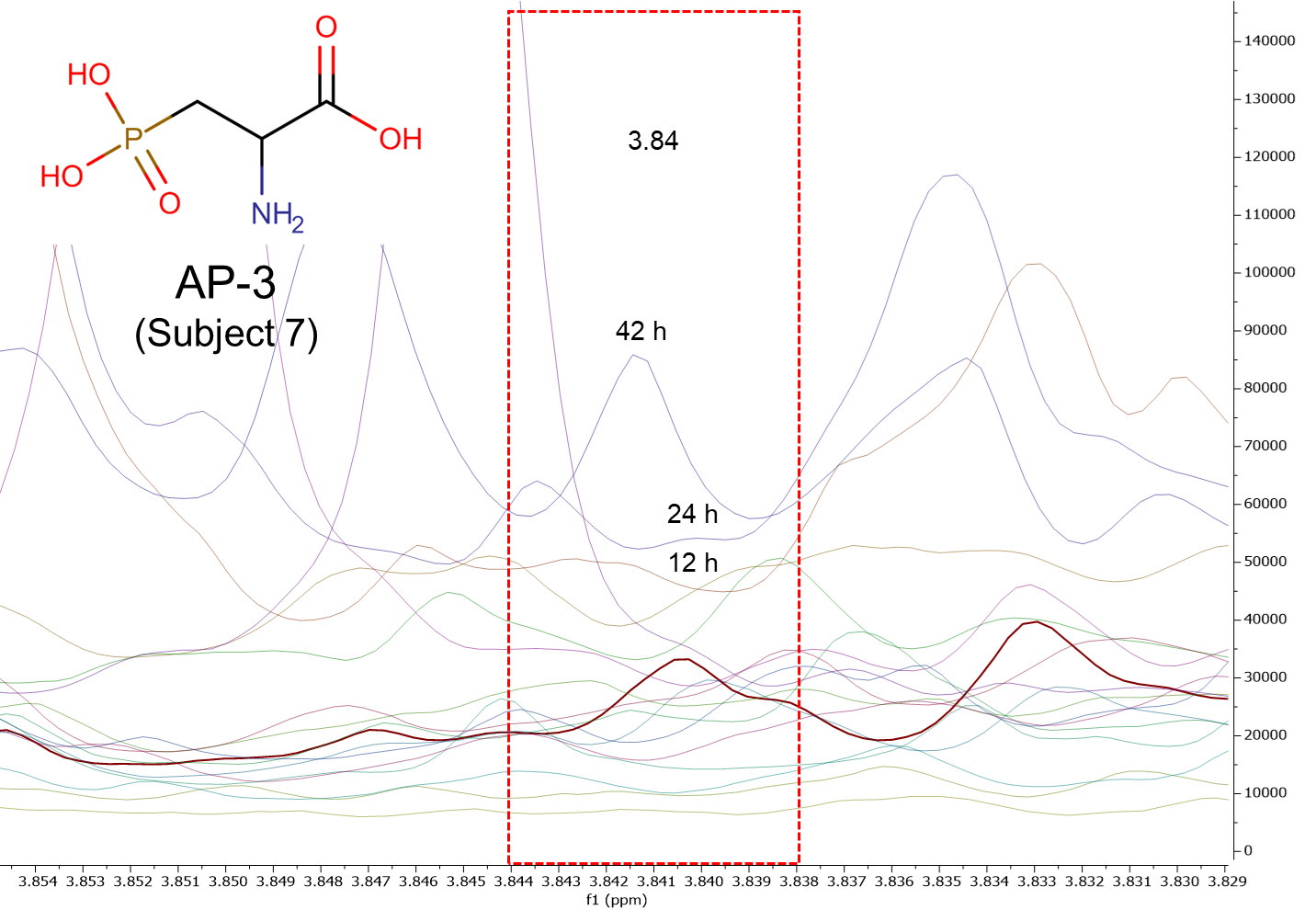


*δ* / ppm

**Figure S2.** Results of NMR-based metabolomics analysis. The 500 MHz ¹H-NMR spectra of 2-amino-3-phos­pho­nopropionic acid (AP-3) in urine samples from subject number 7 are presented. The chromatogram overlay high­lights distinctions between samples collected at different time points, demonstrating changes in the meta­b­olite profile over time. The structure of AP-3 was obtained from the Human Metabolome Database (<https://hmdb.ca/>).


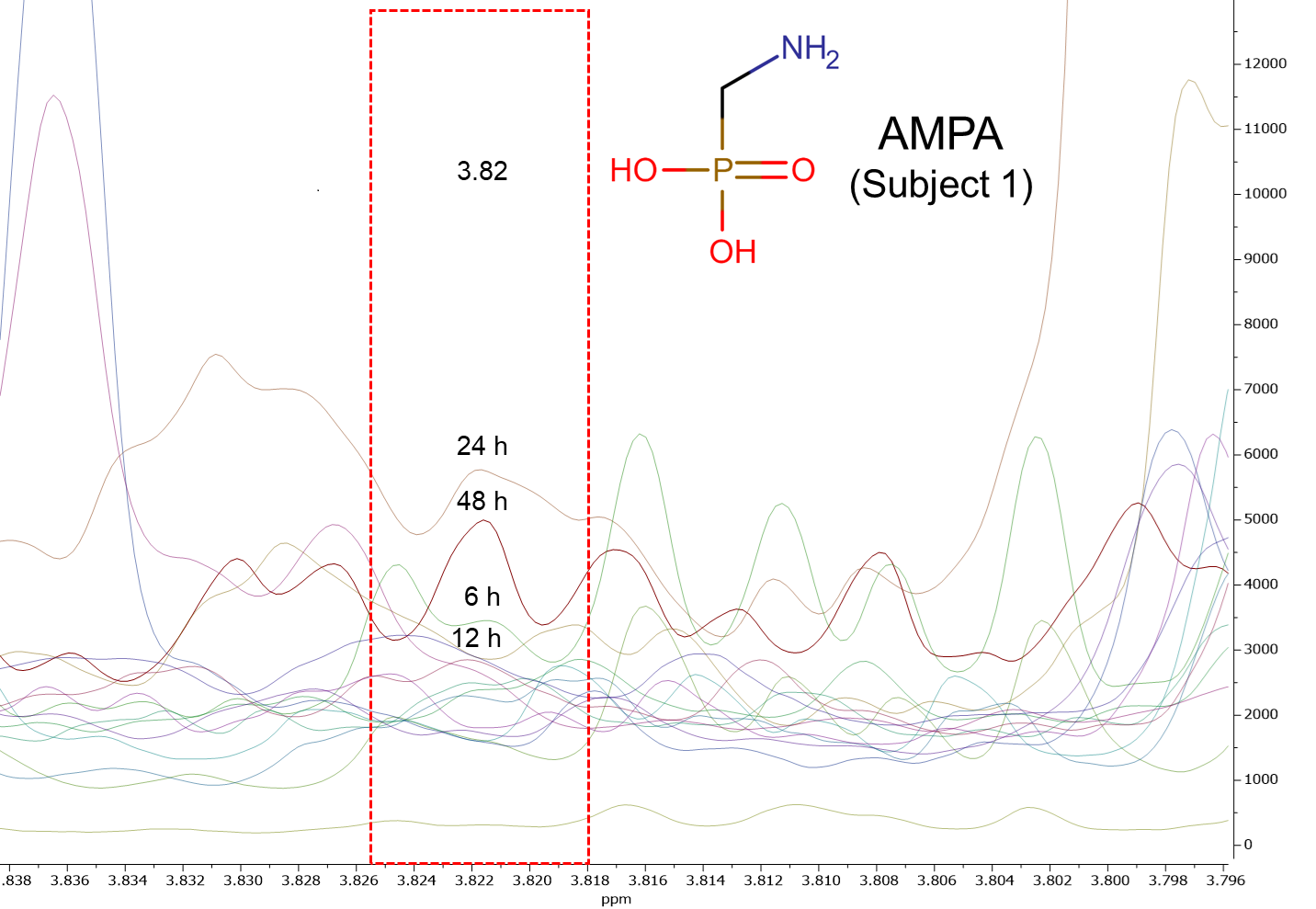


*δ* / ppm

**Figure S3.** Results of NMR-Based Metabolomics Analysis. The 500 MHz ¹H-NMR spectra of aminomethyl­phos­phonic acid (AMPA) in urine samples from subject number 1 are presented. The chromatogram overlay high­lights distinctions between samples collected at different time points, demonstrating changes in the metabolite profile over time. The structure of AMPA was obtained from the Human Metabolome Database (<https://hmdb.ca/>).


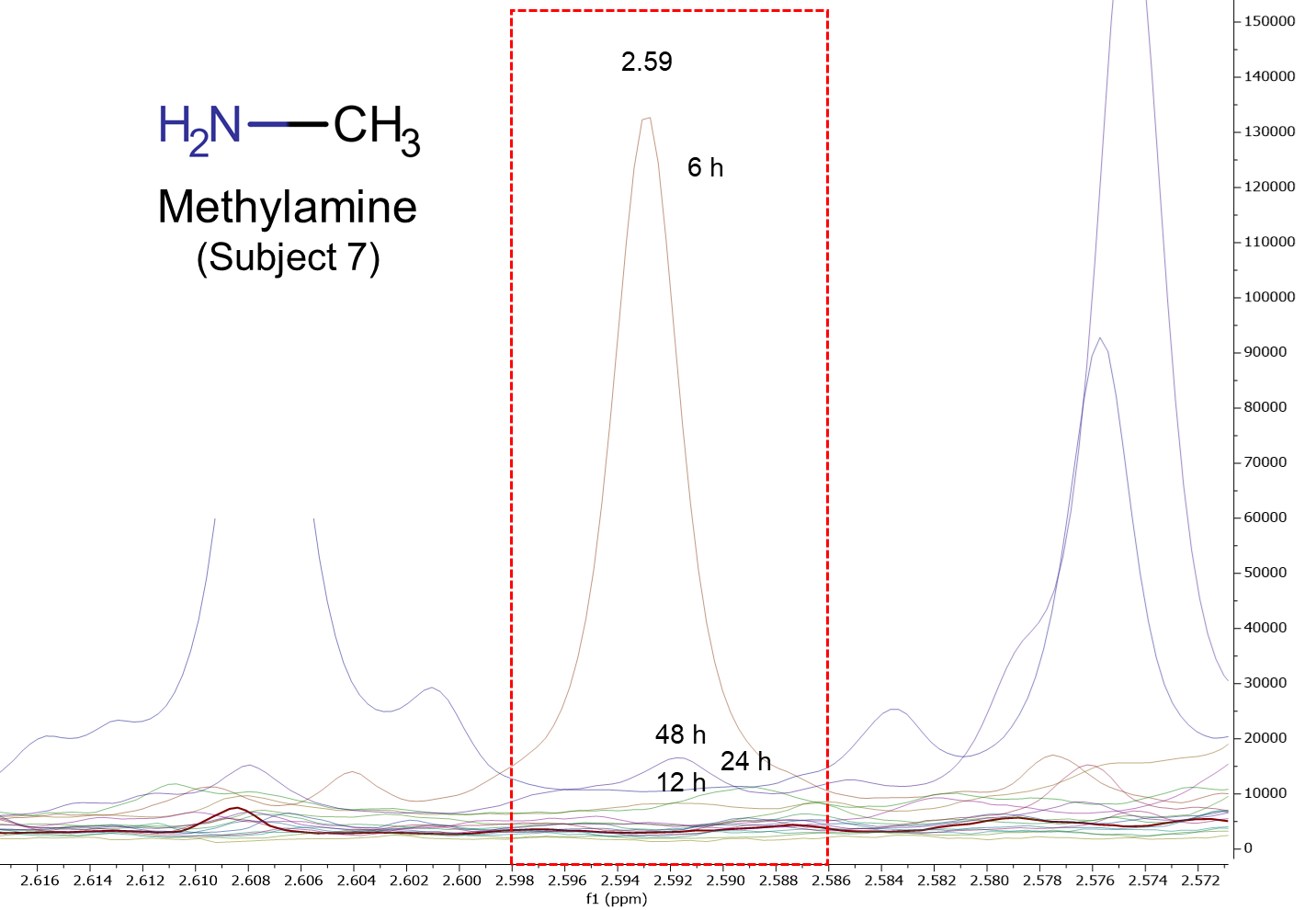


*δ* / ppm

**Figure S4.** Results of NMR-Based Metabolomics Analysis. The 500 MHz ¹H-NMR spectra of methylamine in urine samples from subject number 7 are presented. The chromatogram overlay highlights distinctions between samples collected at different time points, demonstrating changes in the metabolite profile over time. The structure of methylamine was obtained from the Human Metabolome Database (<https://hmdb.ca/>).


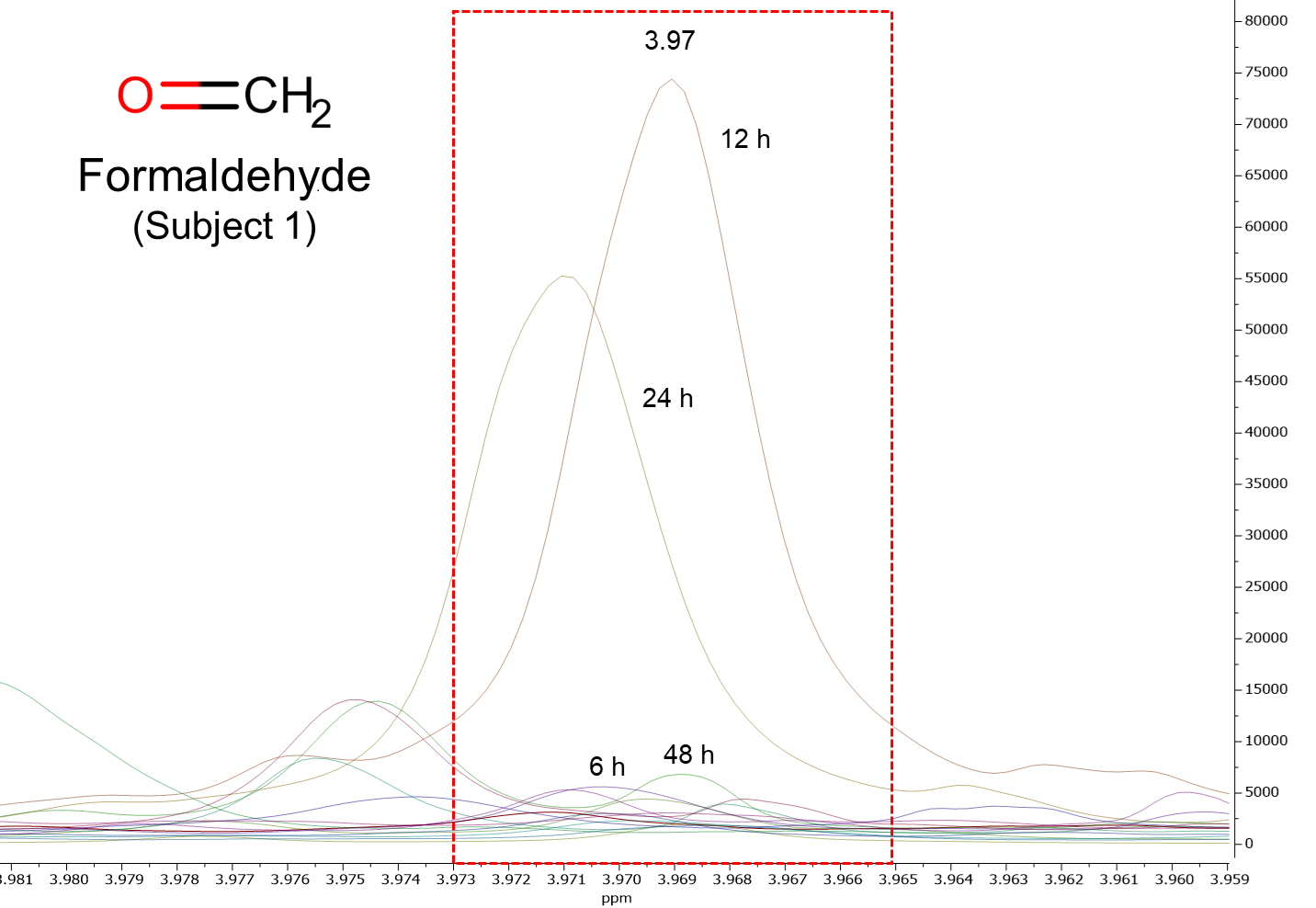


*δ* / ppm

**Figure S5.** Results of NMR-Based Metabolomics Analysis. The 500 MHz ¹H-NMR spectra of formaldehyde in urine samples from subject number 1 are presented. The chromatogram overlay highlights distinctions between samples collected at different time points, demonstrating changes in the metabolite profile over time. The structure of formaldehyde was obtained from the Human Metabolome Database (<https://hmdb.ca/>).


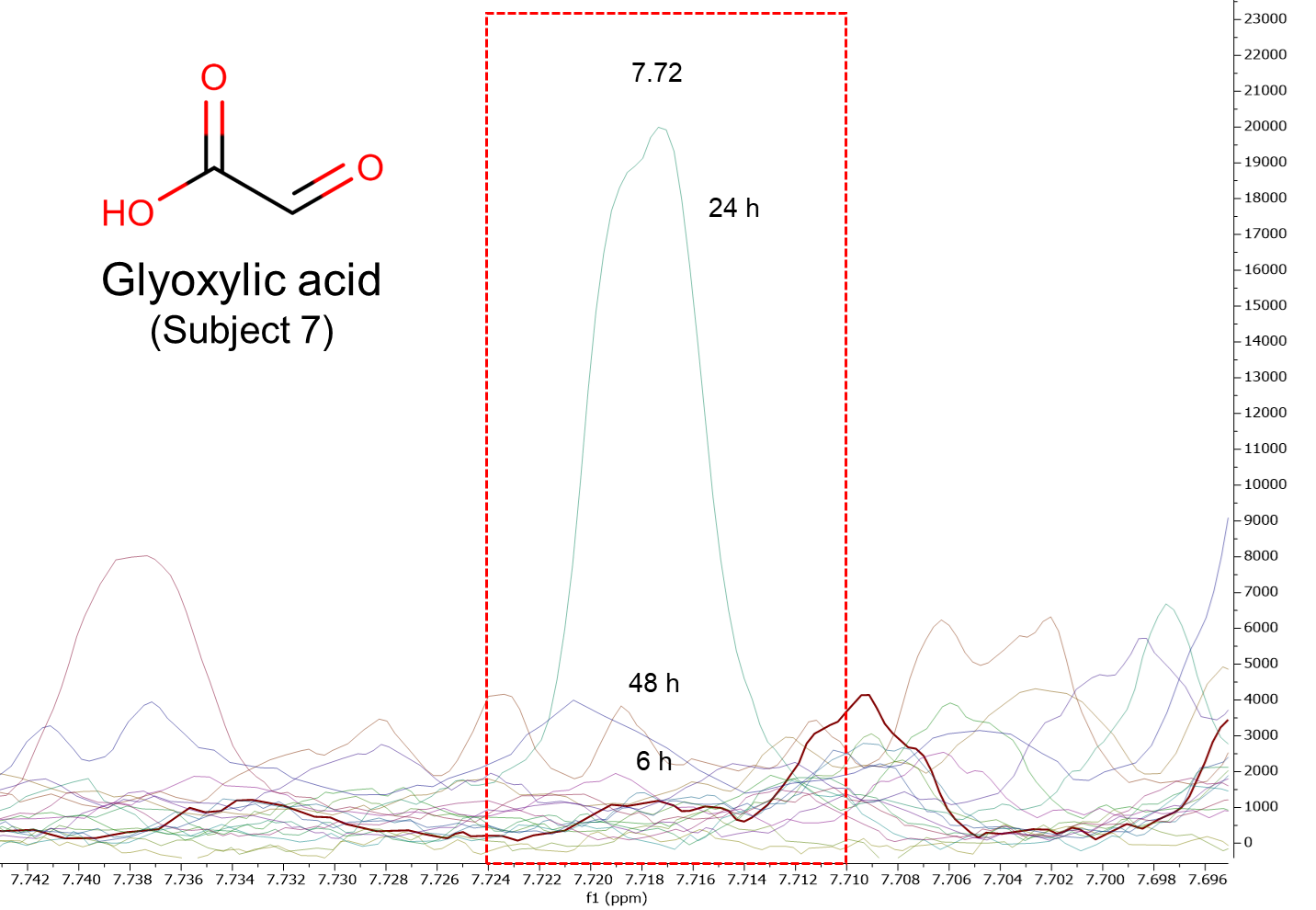


*δ* / ppm

**Figure S6.** Results of NMR-Based Metabolomics Analysis. The 500 MHz ¹H-NMR spectra of glyoxylic acid in urine samples from subject number 7 are presented. The chromatogram overlay highlights distinctions between samples collected at different time points, demonstrating changes in the metabolite profile over time. The structure of glyoxylic acid was obtained from the Human Metabolome Database (<https://hmdb.ca/>).


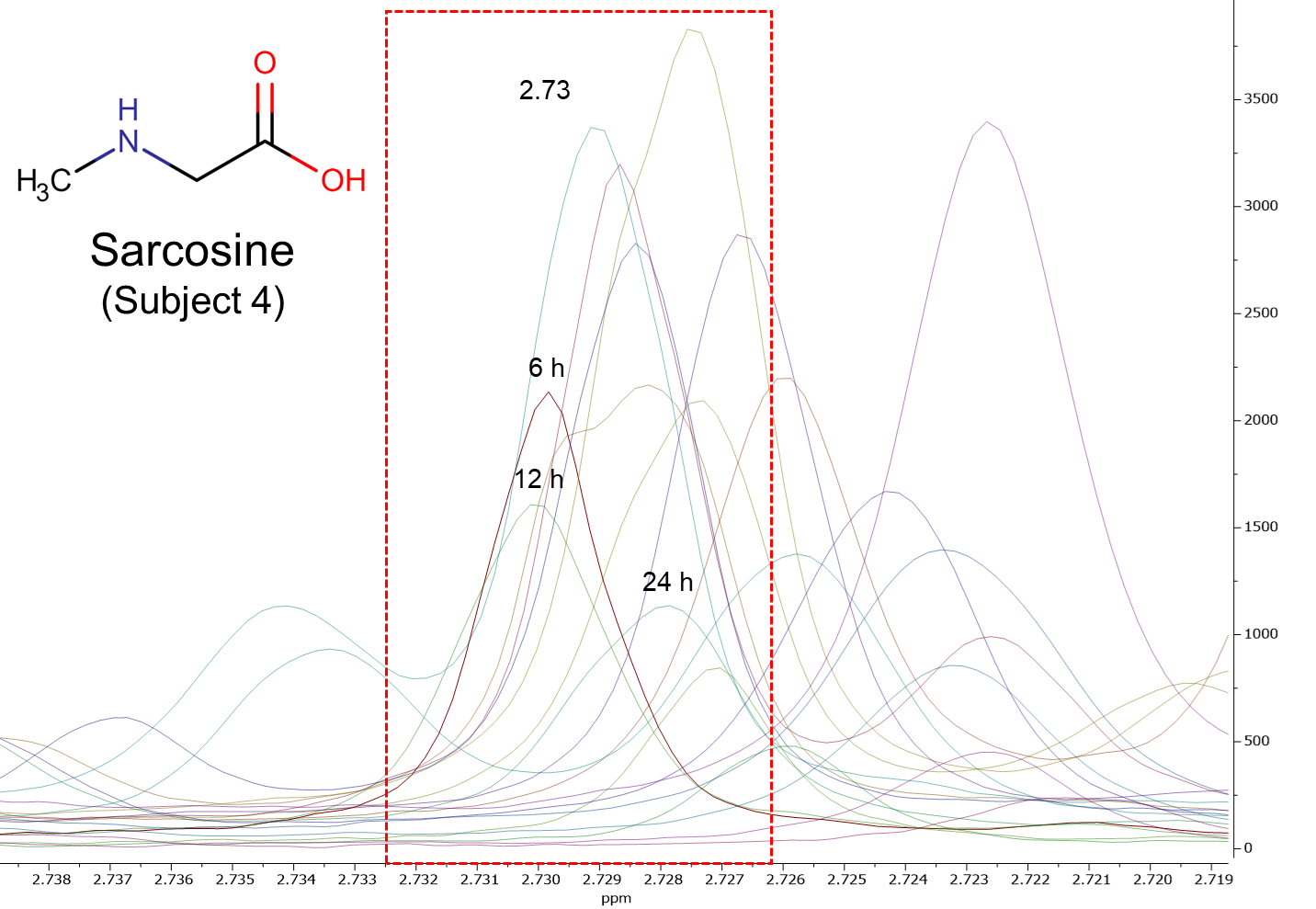


*δ* / ppm

**Figure S7.** Results of NMR-Based Metabolomics Analysis. The 500 MHz ¹H-NMR spectra of sarcosine in urine samples from subject number 4 are presented. The chromatogram overlay highlights distinctions between samples collected at different time points, demonstrating changes in the metabolite profile over time. The structure of sarcosine was obtained from the Human Metabolome Database (<https://hmdb.ca/>).
